# The Treatment of Chronic Bronchitis

**Published:** 1903-04

**Authors:** Robert C. Kenner

**Affiliations:** Louisville, Ky.


### THE TREATMENT OF CHRONIC BRONCHITIS.

BY ROBERT C. KENNER, M.D.,

LOUISVILLE, KY.

There is no more common affection encountered by the general practitioner than chronic bronchitis. The affection is met in varying degrees of advancement, and the prognosis in some instances is as grave almost as phthisis.

Chronic bronchitis, we may observe, is necessarily a resultant affection, and the case which we are called upon to treat may be a mild bronchial inflammation which has existed only a few months, and which carries in its train but little annoyance to the patient, or it will present histories that will carry us back in our search for the cause to exposure in an attack of the measles or other eruptive disease. Other cases present themselves where the patient has taken no systematic treatment, and has dragged on for years with the disease, and is now in a condition almost as serious as a patient with phthisis. In truth, all physicians will readily agree that in many of these patients the results of the disease are the same, whether we are able to find Koch's bacillus in the sputum or not. The treatment of chronic bronchitis is best considered under three heads, first, hygienic treatment; second, treatment of prominent symptoms; third, the administration of agents which exert a curative influence.

In carrying out the first indication, it will be necessary to have our patient dress in such a manner as to thoroughly protect himself against the vicissitudes of the weather. The feet should be kept warm and dry. Warm clothing, and especially clothing which protects the chest, should be worn. I make it a rule to have my patients wear a sleeveless chamois jacket next to the skin, or over the undershirt, on all patients whose occupations entail exposure. The paper vests sold in the drug stores are excellent for ladies and those who only go out occasionally. Attention also to diet is important. Such foods as are known to tax the digestive organs and foods that do not adequately nourish the patient are to be avoided. In a word, much can be done to improve the condition of the patient if attention be paid to these details.

The treatment of prominent symptoms will next claim our attention. Of course these patients come to us for relief of cough. That symptom is the most prominent of all, and will therefore demand especial attention. The administration of cough mixtures will do little to effect a cure in these cases, and the best measures are those which mitigate the severity of the cough. Nearly all cough mixtures contain opium in varying proportions, and upon this agent the efficacy of many cough remedies is dependent. I employ opium for the relief of cough in such cases as are attended with very irritable cough. Some patients will remain awake half of the night coughing if they do not receive something to soothe them. In these cases it is well to give codeine, in doses of a half-grain every hour, until relieved. This remedy is superior to other opiates, since it does not produce constipation and interference with the secretions; yet I never give it for a long period, never longer than a few days. In fact, such agents become useless in most cases if we give well directed remedies to exert a curative influence. These agents we direct the patient to use with caution, and only when the cough is aggravating, or when it keeps him awake, and in that way cause, him loss of strength.

Night sweats and dyspepsia are also complications, or rather prominent symptoms of this affection, which demand attention. Belladonna (the fluid extract) given at bedtime will relieve the night sweats, until, by proper constitutional treatment, we can so reconstruct the patient that he will not have the symptoms.

Dyspepsia is in this and all other affections attended with lowered vital resistance, a symptom which may be exceedingly troublesome. The digestive ferments will often prove efficacious in bringing a cessation of this symptom until the patient has regained strength. Other symptoms may be met and will have to be met on rational grounds. But what can be done in the way of exerting a curative influence on the disease process?

Cod-liver oil formerly was the chief reliance of the profession for the relief of this affection. Yet, all along, many of the ablest and most practical men in the medical profession refused to employ this agent on account of its indigestibility, its disgusting taste, and failure to get good results from it. These and other reasons stand against cod-liver oil, while we have another agent that has none of these drawbacks and is at the same time pleasant to the palate, is a reconstructive, exerts an antiseptic and sedative action on the inflamed tubes, and is a tissue builder of a degree commensurate not surpassed by any agent at our command. The remedy I refer to is Terraline. It is a very palatable preparation of petroleum, and I have employed it for years with happy results. This remedy I give to an adult in doses of one or two teaspoonfuls after each meal. I usually mix this with a wineglass or half wineglass of port or sherry wine. I also have patients to take a dose the last thing on retiring. This makes four doses daily, which soon causes the cough to become looser, less abundant expectorations and the patient soon ceases to have irritation of the tubes, with protracted attacks of coughing.

In some cases we will find that other disease influences may be present, as syphilis, struma, anemia and other factors of a like nature. Manifestly, it is important for the physician to take these factors into account and treat his patient accordingly.

In a word the physician must never fail to weigh carefully all the factors in the case under treatment, and give his patient those remedies which are rationally indicated.

Below, I give in a brief manner, the histories of several patients who have been treated on these lines.

Miss A. Y. W., aged twenty, consulted me for a cough, which had been present for about eighteen months. She had lost flesh very considerably, was pale, weak and low-spirited. She had frequently stayed awake half the night coughing, and every morning had a coughing spell that racked her very much. She had a cough, too, that was characterized by "hacking" and the bringing up of tenacious mucus. Very often she had night sweats, but these were not constant. Her appetite was indifferent and capricious.

She was put on Terraline in doses of two teaspoonfuls in sherry wine after meals and on going to bed. She was given a few powders of codeine, but was told not to use one unless her cough was particularly troublesome.

After using these remedies for ten days she felt improved, and after six weeks her cough had been so mitigated and her general condition so greatly improved that she felt that she might leave off the remedy, but I had her to take it two weeks longer. She is now entirely well and has not taken any medicine for several months.

S. G., aged thirteen, was brought to me for a cough that had been present for two years. She had two years ago suffered with measles, and every fall and winter she suffered with a cough of a most irritating and distressing character. She was put on Terraline in doses of a teaspoonful four times daily. The girl's mother was cautioned to see that her hygienic condition was looked after, and the girl had all the attention possible. On this treatment her cough became gradually less, she gained in flesh, and in a period of. six week was pronounced cured. The girl wore a chamois jacket She never missed school a day.

Mr. J. Y. M., aged twenty-nine, had been a sufferer from bronchitis for two years, and was weak and depleted in flesh and apprehensive lest he was going into consumption. His expectoration was scant and he coughed a great deal at night. He was put on Terraline, two teaspoonfuls four times daily in sherry wine. A chamois jacket was worn over the undershirt, and he was cautioned to observe the best hygienic rules. He was given nothing for the per so. On this treatment he got along well and in three months presented the appearance of a well-nourished man with no cough. He has had no recurrence of symptoms now after a year.

These are only a few of the many cases treated on this principle and were selected at random from my notes.

---

### ANEMIA, IRON AND OIL.

The abundance of fat found in healthy bone marrow and the scarcity of fat in the bone marrow of anemic patients suggests a reason why Cod Liver Oil is so often efficient as a remedy for anemia. Iron certainly acts as a tonic and is an important constituent of hemoglobin but it can scarcely contribute anything towards the organic substance of the red corpuscle. How the bone marrow changes fat into red corpuscles is one of nature's mysteries, but the microscope shows the narrow cells in all stages of transition into new red corpuscles and abundantly surrounded by fat cells.

Cod Liver Oil seems to be a natural food for bone and marrow. Scott's Emulsion, a reliable preparation of Cod Liver Oil, is often of great use in relieving anemic conditions, especially the chlorosis of young women. That anemic blood should regain its color without the administration of iron only reminds us of the fact that ordinary food contains all the iron the system needs and probably the only form of iron the system ever really absorbs.

